# Identification of multiple rare variants associated with a disease

**DOI:** 10.1186/1753-6561-5-S9-S103

**Published:** 2011-11-29

**Authors:** Jeesun Jung, Jessica Dantzer, Yunlong Liu

**Affiliations:** 1 Department of Medical and Molecular Genetics, Indiana University School of Medicine, IB 130, 975 West Walnut Street, Indianapolis, IN 46202, USA; 2 Center for Computational Biology and Bioinformatics, Indiana University School of Medicine, 410 West 10th Street, HS 5000, Indianapolis, IN 46202, USA; 3 Center for Medical Genomics, Fairbanks Hall, Indiana University School of Medicine, 340 West 10th Street, Suite 6200, Indianapolis, IN 46202-3082, USA

## Abstract

Identifying rare variants that are responsible for complex disease has been promoted by advances in sequencing technologies. However, statistical methods that can handle the vast amount of data generated and that can interpret the complicated relationship between disease and these variants have lagged. We apply a zero-inflated Poisson regression model to take into account the excess of zeros caused by the extremely low frequency of the 24,487 exonic variants in the Genetic Analysis Workshop 17 data. We grouped the 697 subjects in the data set as Europeans, Asians, and Africans based on principal components analysis and found the total number of rare variants per gene for each individual. We then analyzed these collapsed variants based on the assumption that rare variants are enriched in a group of people affected by a disease compared to a group of unaffected people. We also tested the hypothesis with quantitative traits Q1, Q2, and Q4. Analyses performed on the combined 697 individuals and on each ethnic group yielded different results. For the combined population analysis, we found that *UGT1A1*, which was not part of the simulation model, was associated with disease liability and that *FLT1*, which was a causal locus in the simulation model, was associated with Q1. Of the causal loci in the simulation models, *FLT1* and *KDR* were associated with Q1 and *VNN1* was correlated with Q2. No significant genes were associated with Q4. These results show the feasibility and capability of our new statistical model to detect multiple rare variants influencing disease risk.

## Background

The identification of common variants associated with a disease has been successful through the use of genome-wide association studies (GWAS). However, most of the associated single nucleotide polymorphisms (SNPs) have small effect sizes and small proportions of heritability [1]. In addition, some GWAS have failed to detect disease causal variants because of the strong assumption that common variants contribute to an increase in risk of common diseases (the common disease/common variant hypothesis) [2]. Recently several rare variants have been identified that confer a substantial risk for autism, mental retardation, and schizophrenia [1]. These observations support a hypothesis that rare variants could be the primary drivers of common diseases (the common disease/rare variant hypothesis). This hypothesis assumes that a significant proportion of the inherited susceptibility to relatively common human disease may be caused by the accumulation of the effects of a series of low-frequency variants acting dominantly or additively to increase the relative risk for disease [2].

GWAS have been designed to achieve statistical power for variants occurring in more than 5% of the general population, and they provide little information about relatively common variants with frequencies between 1% and 5%. However, recent advances in next-generation sequencing technologies and ventures, such as the 1000 Genomes Project, allow for the introduction of novel rare variants that most likely occur in less than 5% (or even in less than 1%) of one or more major human populations. Although knowledge of these novel rare variants can be used in association studies of common diseases, statistical analyses are challenging because the ordinary SNP-by-SNP methods that are suited for GWAS have limited capacity to detect rare variant association because of the extremely low frequency of each variant [3]. Furthermore, statistical power is dramatically reduced when we take into account correction for multiple tests. Therefore one of the key challenges in rare variant association studies is how to capture (i.e., group) the variants by genomic region to overcome the reduction in power experienced in ordinary SNP-by-SNP methods.

In this paper, we collapse rare variants within a gene in two ways: first, using rare variants of all SNPs, and, second, using only rare variants of nonsynonymous SNPs to see the functional effect on disease traits. We then test for association of the rare variants with disease traits under the hypothesis that the number of rare variants within a gene is correlated either positively or negatively with the traits. To perform this test, we apply a novel statistical approach, called zero-inflated Poisson regression models, which provides flexibility for the excess of zeros caused by the extremely low frequency of the variants [4]. We test 3,205 genes under two scenarios: one including a single group made up of all 697 subjects after adjusting for population substructure and the other involving separating the subjects into three ethnic groups based on principal components analysis and geographic information. Results from these analyses show the feasibility of using this new statistical model to take into account the excess of zeros and to detect multiple rare variants responsible for disease risk.

## Methods

### Data

The genotypes for 24,487 exonic SNPs from 3,205 genes included in the 1000 Genomes Project were distributed by Genetic Analysis Workshop 17 (GAW17) [5]. The SNPs consist of synonymous SNPs and nonsynonymous SNPs. Data for 697 individuals was provided for analysis and consisted of seven population groups: European Americans, Tuscans (from Italy), Yoruba, Luhya (from Kenya), Han and Denver Chinese, and Japanese. For phenotypes, 200 replicate simulations were carried out to give disease status (1 = affected, 0 = not affected) and three quantitative traits (Q1, Q2, Q4) for each individual. Information such as smoking status, sex, and age of each individual was also available.

### Collapsing rare variants

Analysis of single SNPs with extremely low frequency leads to a reduction in statistical power, especially when correcting for multiple comparisons. To overcome this obstacle, we consider collapsing multiple rare variants into a single gene-based variant. The advantages of this strategy are that it reduces the number of tests and that it enhances the variability of the variants as mutational signals [6]. Assume that *N* is the total number of individuals, *m* is the total number of genes, and *n_j_* is the total number of SNPs on the *j*th gene. We define an indicator variable for the genotype of the *k*th SNP of the *j*th gene for the *i*th individual as follows:(1)

where *a* is the minor allele of the *k*th SNP, *i* = 1, …, *N*, *j* = 1, …, *m*, and *k* = 1, …, *nj*. This indicator variable describes the presence of a rare variant equivalently to:(2)

We then sum *V_ijk_* over all SNPs (*k* = 1, 2, …, *n_j_*) on the *j*th gene:(3)

### Statistical models

For each gene, we applied a zero-inflated Poisson model based on the results of collapsing the variants. Because our method is a gene-based approach, subscript *j* is omitted for convenience. The statistical models are:(4)

where:(5)

is the total number of rare variants at the *j*th gene for the *i*th subject. Parameters *p* = (*p*_1_, *p*_2_, …, *p_N_*)*^T^* and *μ* = (*μ*_1_, *μ*_2_, …, *μ _N_*)*^T^* are modeled by means of canonical link generalized linear models as:(6)(7)

where *X_i_* is an indicator variable given by *X_i_* = 1 for a case individual and *X_i_* = 0 for a control individual in each replicate data set and *Z*_1_*_i_*, *Z*_2_*_i_*, and *Z*_3_*_i_* are age, sex, and smoking status, respectively. PCS_ip_ is the principal component score obtained by multidimensional scaling (MDS) analysis of identical-by-state pairwise distances in PLINK. For a quantitative trait analysis, the statistical model uses the same framework but replaces *X_i_* with the quantitative trait (Q1, Q2, Q4) value for each subject. We test *β*_1_ = 0 for the association of each trait. All statistical analyses were performed using SAS software.

### Analysis procedure

Association studies are sensitive to population stratification. The results often vary as a result of differences in minor allele frequency, linkage disequilibrium patterns, causal pathways, or environmental exposures. To minimize the effects of such variability, we must examine the relationship between genetic variants and disease traits within ethnicities. For the GAW17 data set we analyzed, we performed an MDS analysis based on all the available exonic SNPs using PLINK to estimate population substructure and to detect outliers. The results of the MDS analysis classified individuals as belonging to one of three major ethnic groups: European Americans and Tuscans in Italy were grouped together as Europeans, Yoruba in Nigeria and Luhya in Kenya were grouped as Africans, and Japanese and Chinese in both Beijing and Denver were grouped as Asians. These populations were both geographically and genetically similar. In addition, we performed principal components analysis on all exonic SNPs using Eigenstrat [7] and obtained the first 10 main eigenvectors, which can track membership in populations with 99.9% accuracy [7]. We control the 10 eigenvectors for subtle subpopulation stratification caused by the seven original populations in the statistical model.

We then performed genotype-phenotype association analysis under two scenarios: (1) analyzing the three ethnic groups separately to account for different minor allele frequencies among the SNPs and (2) analyzing all 697 individuals together in one large group to compare the results. We adjusted for the first 10 eigenvectors in both cases to control for subtle subpopulations, as noted previously. For each subject in a given group, we first indicated the presence of the minor allele for each SNP and then collapsed multiple rare variants within each gene for both the all-exonic-SNPs model and the nonsynonymous-SNPs-only model, as previously described. We applied the zero-inflated Poisson model to identify significant genes associated with the disease trait and quantitative traits. *p*-values for 3,205 autosomal genes were obtained from each analysis. The estimated *p*-value threshold was 1.56 × 10^−5^ (= 0.05/3,205) after controlling for multiple tests. Next, we analyzed the 200 replicates provided in the data set to calculate the power at a significance level of 0.05 and 1.56 × 10^−5^ in order to compare our results with the true genes in the GAW17 answers.

## Results

MDS analysis revealed three ethnic groups formed by the seven populations in the data set. Five outliers were found among the 697 subjects and were subsequently removed, leaving the total number of individuals used in the analysis at 692. Broken down by ethnic group, we found 215 Africans, 321 Asians, and 156 European individuals (data not shown) [7]. To determine whether genetic structure differed across the ethnic groups, we estimated the minor allele frequencies of the SNPs found on two randomly selected genes. Table 1 shows how the minor allele frequencies and rare variants found in each gene vary greatly across the three ethnic groups. To account for these differences, we collapsed the rare variants within a gene and performed the analysis for each ethnic group separately.

**Table 1:** Minor allele frequencies of SNPs within two randomly selected genes

Gene	Chromosome	SNP	Function	Minor allele	Minor allele frequency			
					
					All*	Europeans	Asians	Africans
*CFH*	1	C1S8678	Nonsynonymous	A	0.341	0.032	0.579	0.982
		C1S8682	Synonymous	C	0.001	0	0	0.005
		C1S8684	Synonymous	A	0.311	0.923	0.106	0.964
		C1S8686	Nonsynonymous	C	0.325	0.942	0.134	0.959
*SMAD7*	18	C18S2642	Synonymous	C	0.006	0	0.022	0
		C18S2643	Synonymous	A	0.009	0	0.034	0
		C18S2648	Synonymous	T	0.006	0	0.028	0

* All 697 individuals were used to calculate the minor allele frequency.

Table 2 shows genes that were significantly associated with disease liability and quantitative traits Q1, Q2, and Q4 based on the pooled data set of 692 individuals. For each of these genes, the table displays the percentage of replicates, out of 200, whose *p*-values were less than 0.05 before adjusting for multiple tests or less than 1.56 × 10^−5^ after adjusting for multiple tests. Genes that were significant at the 5% significance level in at least 70% of replicates are displayed in Table 2. Genes in boldface are causal genes in the simulation model, and those not in boldface are examples of false-positive association that were seen frequently across replicates.

**Table 2:** Genes associated with disease liability, Q1, and Q2 for the pooled populations at the significance levels of 0.05 and 1.56 × 10^−5^

Trait	All SNPs (nonsynonymous and synonymous)			Nonsynonymous SNPs		
	
	Gene	Replicates significant at 0.05 (%)^a^	Replicates significant at 1.56 × 10^−5^ (%)^b^	Gene	Replicates significant at 0.05 (%)^a^	Replicates significant at 1.56 × 10^−5^ (%)^b^
Disease liability (case-control)	*CYSLTR2*	77.5	68.5	*AKR1D1*	72.5	36.5
	*MGEA5*	84	79.5	*AQP3*	74	66.5
	*NF2*	85.5	76.5	*CAPNS1*	74	64
	*PTPN11*	83.5	71	*EIF6*	84	77.5
	*UGT1A1*	72.5	55	*FLT1*	72.5	5
				*LYPD6*	77.5	72.5
				*PCDHGA4*	77	69.5
				*PRKCG*	70	65
				*UGT1A1*	71.5	55.5

Q1	*CYSLTR2*	62.5	45	***FLT1***	96.5	97.5
	***FLT1***	100	92	*SLC2A13*	69.5	0
	*PTPN11*	64.5	42.5			

Q2	*PIK3R1*	66.5	29	***VNN1***	62	0
	*PTGIS*	62.5	41.5			
	*PTPN11*	71.5	51.5			

Listed genes were significant in at least 70% of replicates. Genes in boldface are the true causal genes in the simulation model.

^a^ Percentage of replicates in which the gene was significantly associated with disease liability, Q1, or Q2 at the significance level of 0.05.

^b^ Percentage of replicates in which the gene was significantly associated with disease liability, Q1, or Q2 at the significance level of 1.56 × 10^−5^

Through the disease liability analysis, we identified four unique genes with a replication rate of more than 70% when using all exonic SNPs and eight unique genes when using only nonsynonymous SNPs. *UGT1A1*, which was not part of the simulation model, was detected in both cases. Analysis of quantitative traits Q1 and Q2 identified five unique genes when including all exonic SNPs and two unique genes when including only nonsynonymous SNPs. *FLT1*, one of the true causal genes in the simulation model, was associated with Q1 in both cases with a replication rate of more than 90% after adjusting for multiple tests. *PTPN11*, which was not a true causal gene in the model, was identified in association with disease liability, Q1, and Q2 when analyzing all exonic SNPs but not when analyzing only nonsynonymous SNPs. Also, based on a replication rate criterion of 70%, we found no significant genes associated with Q4 in any of the scenarios.

Table 3 lists the genes that are significantly associated with disease liability for each ethnic group when using all exonic SNPs. As illustrated, many of the genes that were significantly associated in a high percentage (>70%) of replicates in one particular ethnic group were not detected in the combined sample. All these associations were false-positive results. Tables 4 and 5 provide the power to detect significant association of genes that were in the GAW17 simulation models. Three genes (*FLT1* for Q1 and disease liability, *KDR* for Q1, and *VNN1* for Q2) were detected with moderate to high power in either the all-exonic-SNPs or the nonsynonymous-SNPs-only analysis. *PTK2* was associated with disease liability with a power of 50% in the nonsynonymous-SNPs-only analysis.

**Table 3:** Genes significantly associated with disease liability for each ethnic group at the significance levels of 0.05 and 1.56 × 10^−5^

Africans (*n* = 215)			Europeans (*n* = 156)			Asians (*n* = 321)		
Gene	Replicates significant at 0.05 (%)^a^	Replicates significant at 1.56 × 10^−5^ (%)^b^	Gene	Replicates significant at 0.05 (%)^a^	Replicates significant at 1.56 × 10^−5^ (%)^b^	Gene	Replicates significant at 0.05 (%)^a^	Replicates significant at 1.56 × 10^−5^ (%)^b^

*CYSLTR2*	84	71	*CCNH*	72	57	*CHEK1*	95.5	95.5
*FER*	77	50.5	*CLPTM1*	71	38	*FLJ43860*	72.5	64
*FNDC3A*	73	68	*CYP17A1*	74	65.5	*IL4I1*	74.5	53.5
*NTRK1*	71	59.5	*GDNF*	83	22	*LRRC20*	74.5	69.5
*OR8J3*	71.5	51.5	*OR13A1*	86.5	82.5	*NF2*	87.5	67.5
*PTPN11*	86.5	74				*SPINK2*	89.5	47
						*ST5*	84.5	79.5

Listed genes were significant in at least 70% of replicates. None of the genes were true causal genes in the simulation model. Analysis is for all SNPs.

^a^ Percentage of replicates in which the gene was significantly associated with disease liability at the significance level of 0.05.

^b^ Percentage of replicates in which the gene was significantly associated with disease liability at the significance level of 1.56 × 10^−5^.

**Table 4:** Power to detect causal genes (out of 35 genes) in the GAW17 simulation models using the combined sample

Trait	All SNPs (nonsynonymous and synonymous)			Nonsynonymous SNPs		
	
	Gene	Power calculated at significance level 0.05 (%)	Power calculated at significance level 1.56 × 10^−5^ (%)	Gene	Power calculated at significance level 0.05 (%)	Power calculated at significance level 1.56 × 10^−5^ (%)
Disease liability	*FLT1*	100	92	*PTK2*	54.5	33.5
				FLT1	96.5	97.5

Q1	*FLT1*	100	92	*FLT1*	96.5	97.5
	*KDR*	56.5	0	*KDR*	54.5	0

Q2	*VNN1*	46.5	0	*VNN1*	62	0

**Table 5:** Power to detect causal genes in the GAW17 simulation model as associated with disease liability using each ethnic group

Africans (*n* = 215)			Europeans (*n* = 156)			Asians (*n* = 321)		
Gene	Power calculated at significance level 0.05 (%)	Power calculated at significance level 1.56 × 10^−5^ (%)	Gene	Power calculated at significance level 0.05 (%)	Power calculated at significance level 1.56 × 10^−5^ (%)	Gene	Power calculated at significance level 0.05 (%)	Power calculated at significance level 1.56 × 10^−5^ (%)

*PIK3C3*	58	32	*PIK3C3*	66.5	51.5	*NRAS*	43	33
			*SHC1*	42	29.5			

Analysis is for all SNPs.

## Discussion and conclusions

It is well known that not all coding rare variants within coding portions of genes are causative, and grouping them together regardless of their functional effect may produce false-positive errors. Therefore successful use of collapsing strategies increases the power to detect potential causative variants in association studies. We have applied a zero-inflated Poisson regression model to two separate collapsing methods: one in which we used all available exonic SNPs and the other in which we limited the data to nonsynonymous SNPs alone. Based on these analyses, we found that four unique genes were associated with disease liability and five were in association with quantitative traits Q1 and Q2 when collapsing all exonic SNPs within a gene. When collapsing only the nonsynonymous SNPs within a gene, we found eight unique genes in association with disease liability and two unique genes from Q1 and Q2. *UGT1A1* and *FLT1* were the only genes detected by both analyses. The differences between these results show how the detection of rare variant association depends on the collapsing strategy used.

In addition to differences in the collapsing strategy, our approach to analyzing each ethnic group versus the combined sample group shows how variations in population can affect the results. Six genes on average were detected from each ethnic group. Few of the same genes, whether they were false-positive associations or true associations, were detected by the analysis of the combined sample group versus the individual ethnic groups. From the results of our analyses, we can also see that the three ethnic groups have a wide range of allele frequencies for some SNPs. Variants within the same gene that have different allele frequencies should not be collapsed into the same group to avoid a loss of statistical power. The GAW17 simulation model did not take population allele frequency differences into account, and this could be the cause of much of the discrepancy in the detection of genes using various collapsing approaches or analytic strategies. Therefore ethnicity-specific analysis might be a better approach to performing association studies of genome sequence data than using the full combined sample group because causal rare variants may have different frequencies in different ethnic groups in real human populations.

In using our novel approach to identify rare variants associated with disease traits, we found that 5 causal genes (out of 35) in the simulation models were detected with a reasonable power. However, we did not detect many of the genes in the GAW17 answers as being significant, for several reasons. First, our statistical model does not take into account any pathway information. In the simulation model the genes associated with Q1 are from the vascular endothelial growth factor (VEGF) pathway, and the genes associated with Q2 are from the cardiovascular disease risk and inflammation pathways, both of which influence disease liability. The power to detect true causal variants might have been improved if all rare variants within a pathway were collapsed together into a single pathway variant indicator. Second, the effect size of many of the “causal” variants was quite small, thereby limiting power. Third, the assumptions made by a zero-inflated Poisson model for the collapsed rare variants might not be suitable for some genes with only a few SNPs. In that case, one might need to apply a zero-inflated binomial regression to detect the association, or one may need to take into account the dispersion parameter of variance in the model for consistent parameter estimation. Therefore further research is needed to evaluate the zero-inflated Poisson regression model for its ability to handle the various characteristics of various rare variants.

## Competing interests

The authors declare that there are no competing interests.

## Authors’ contributions

JJ developed statistical models, performed the analysis and wrote the manuscript. JD helped data formatting and English correction in the manuscript. YL contributed on the interpretation of the analysis.
